# Comparative Effectiveness of Finerenone Versus SGLT2 Inhibitors in Patients with HFpEF and CKD: A Real-World Propensity-Matched TriNetX Analysis

**DOI:** 10.3390/biomedicines14051108

**Published:** 2026-05-14

**Authors:** Faizan Ahmed, Saifullah Khan, Najam Gohar, Fenilkumar Kotadiya, Muhammad Hassan, Nisha Khalid, Muhammad Hussain, Tehmasp Rehman Mirza, Faseeh Haider, Muhammad Abdullah, Haris Bin Tahir, Mohammad Hamza, Ameer Haider Cheema, Amro Taha, Mohammad Omar Butt, Shamaiza Waqas, Fawaz Alenezi

**Affiliations:** 1 Department of Internal Medicine, Hackensack Meridian Health, Jersey Shore University Medical Center, Neptune, NJ 07753, USA; faizan.ahmed1@hmhn.org; 2 Department of Internal Medicine, Dow Medical College, Dow University of Health Sciences, Karachi 74200, Sindh, Pakistan; saifrah456@gmail.com (S.K.); azamh5223@gmail.com (M.H.); nishakhalid.2405@gmail.com (N.K.); azamhhh7@gmail.com (M.H.); 3 Department of Internal Medicine, Ameeruddin Medical College, PGMI, Lahore 54000, Punjab, Pakistan; najamgoharr@gmail.com; 4 Department of Internal Medicine, Charleston Area Medical Center, Charleston, WV 25301, USA; drfenilkotadiya@gmail.com; 5 Department of Internal Medicine, Shalamar Medical and Dental College, Lahore 54840, Punjab, Pakistan; tehmaspmirza@gmail.com (T.R.M.); mohd_abdullah2003@outlook.com (M.A.); 6 Department of Internal Medicine, Allama Iqbal Medical College, Lahore 54550, Punjab, Pakistan; 7 Department of Internal Medicine, Lahore General Hospital, Lahore 54000, Punjab, Pakistan; harristahirchh@gmail.com; 8 Department of Internal Medicine, Albany Medical Center, Albany, NY 12208, USA; mohammad_hamza_7789@hotmail.com; 9 Department of Internal Medicine, University of Texas Southwestern Medical Center, Dallas, TX 75390, USA; ameerhaider.cheema@utsouthwestern.edu; 10 Division of Cardiology, West Virginia University, Morgantown, WV 26506, USA; taha61591@gmail.com; 11 Department of Internal Medicine, Memorial Satilla Health, Waycross, GA 31501, USA; omar9224@gmail.com; 12 Department of Internal Medicine, Corewell Health, University Hospital, Royal Oak, MI 48073, USA; shamaizawaqas@gmail.com; 13 Division of Cardiology, Duke University Hospital, Durham, NC 27710, USA; fawaz.enezi@duke.edu

**Keywords:** HFpEF, chronic kidney disease, finerenone, SGLT2 inhibitors, propensity score matching

## Abstract

**Background**: Finerenone and sodium–glucose cotransporter-2 inhibitors provide cardiovascular and renal benefits in patients with chronic kidney disease (CKD) and heart failure (HF), but real-world comparative evidence is limited. **Methods**: This retrospective study used the TriNetX database. Adults ≥ 40 years with CKD stages 1–5 and HF [LVEF > 40%; excluding end-stage renal disease (ESRD) or dialysis] receiving finerenone were compared with those on SGLT2 inhibitors. Propensity score matching (1:1) yielded 333 patients per cohort. Kaplan–Meier and Cox models estimated hazard ratios (HRs) with 95% confidence intervals. **Results**: After matching, baseline characteristics were reasonably balanced, with some residual imbalance remaining. All-cause mortality was similar between finerenone and SGLT2 inhibitors at 6 months (HR 0.98; 95% CI 0.50–1.90) and 1 year (HR 0.93; 95% CI 0.53–1.66). All-cause hospitalization or ER visits were also comparable at 6 months (HR 1.07; 95% CI 0.84–1.36) and 1 year (HR 1.04; 95% CI 0.83–1.29). Finerenone was associated with a modest, borderline reduction in HF hospitalization at 1 year, without consistent effects across timepoints or a mortality benefit; thus, this finding is hypothesis-generating (HR 0.81; 95% CI 0.66–0.99). Safety outcomes were similar between groups. **Conclusions**: In this real-world analysis, finerenone was associated with similar all-cause mortality, overall hospitalization, and renal safety outcomes compared with SGLT2 inhibitors, with a modest reduction in HF hospitalization at 1 year that should be interpreted cautiously given the exploratory nature of the study. These findings are hypothesis-generating and underscore the need for prospective head-to-head trials to better define optimal therapy sequencing in patients with HFpEF and CKD.

## 1. Introduction

Heart failure with preserved ejection fraction (HFpEF) represents nearly half of all heart failure (HF) cases and is expected to emerge as the predominant HF phenotype by 2030, driven largely by population aging and the increasing burden of cardiometabolic comorbidities [1,2,3]. Chronic kidney disease (CKD) is defined by evidence of kidney damage or an estimated glomerular filtration rate (eGFR) < 60 mL/min/1.73 m^2^ persisting for ≥3 months, often accompanied by albuminuria (>30 mg/g). As renal function progressively declines, cardiovascular structural abnormalities become increasingly prevalent, particularly left ventricular hypertrophy, which affects more than two-thirds of patients by the time kidney replacement therapy (dialysis or transplantation) is required. In this population, HFpEF emerges as one of the most common—and potentially dominant—cardiovascular disease phenotypes [4,5]. Addressing this high-burden intersection remains a critical unmet need in real-world practice.

SGLT2 inhibitors represent the first clearly effective disease-modifying therapy for HFpEF. Evidence from recent randomized trials and complementary meta-analyses has consistently shown their robust cardioprotective and renoprotective benefits across a broad spectrum of patients with HF and CKD, with the magnitude of absolute clinical benefit clearly outweighing potential risks [6]. Current literature suggests that finerenone, a non-steroidal mineralocorticoid receptor antagonist, offers significant benefits in patients with HFpEF and CKD, especially those with type 2 diabetes. It reduces HF hospitalizations, slows disease progression—including in older adults—and lowers all-cause mortality [7,8]. Compared with steroidal MRAs, finerenone has a safer profile, with fewer electrolyte and renal complications, making it suitable for patients with complex comorbidities [7,8].

Despite robust evidence supporting finerenone, SGLT2 inhibitors, and other therapies in patients with HFpEF and CKD, the optimal first-line cardiorenal strategy remains uncertain. No head-to-head randomized trials have directly compared finerenone with SGLT2 inhibitors, and differences in trial populations and designs limit indirect comparisons. Consequently, clinicians lack clear guidance on relative efficacy for reducing HF hospitalizations, slowing renal progression, or selecting the optimal sequence of therapy initiation. This uncertainty is further compounded by limited real-world comparative effectiveness and safety data across diverse CKD-HFpEF populations, leaving critical knowledge gaps in high-risk patients.

To address these knowledge gaps, we conducted a real-world comparative effectiveness study evaluating clinical outcomes of finerenone versus SGLT2 inhibitor therapy in patients with HFpEF and CKD. Leveraging a large, federated electronic health record database and PSM cohorts, we examined differences in all-cause mortality, HF hospitalizations, overall hospitalization, and renal safety outcomes.

## 2. Methods

### 2.1. Data Source

This observational study was conducted by using the TriNetX Research network, a global federated platform offering access to de-identified electronic medical records (EMRs). The query for this data analysis was generated on 1 March 2026 which drew from a diverse pool of 171 healthcare organizations (HCOs) within the Global Collaborative Network. TriNetX network aggregates patient-level clinical data, including medications, diagnoses, and lab results in accordance with the Health Insurance Portability and Accountability Act (HIPAA). As only de-identified and aggregated information was used, this study was considered exempt from institutional review board (IRB) oversight.

### 2.2. Study Population and Design

The study included patients aged ≥40 years diagnosed with CKD stages 1–5 (or unspecified) alongside a history of HFpEF, including diastolic, other, or unspecified HF phenotypes. Individuals were excluded if they had a left ventricular ejection fraction (LVEF) ≤40%, systolic HF, ESRD, or dependence on renal dialysis. HFpEF was identified using ICD-10 diagnostic codes for diastolic heart failure (I50.3), other heart failure (I50.8), and heart failure, unspecified (I50.9), in patients without any recorded diagnosis of reduced ejection fraction (LVEF ≤ 40%). While the inclusion of ‘unspecified heart failure’ codes may introduce heterogeneity, this approach was adopted to improve sensitivity and capture real-world clinical practice patterns, where HF phenotype is not always consistently coded. To partially mitigate misclassification, patients with documented reduced ejection fraction were excluded. Nevertheless, this operational definition may include a mixed population encompassing HFpEF, HFmrEF, and patients with recovered ejection fraction, and should be interpreted accordingly. Cohorts were constructed using standardized diagnostic and medication identifiers available within the TriNetX Research Network database. CKD stages, HF phenotypes, and exclusion conditions were identified using relevant ICD-10-CM diagnostic codes, while medication exposure to Finerenone and SGLT2 inhibitors was defined using RxNorm and Anatomical Therapeutic Chemical (ATC) classifications. A comprehensive list of diagnostic and medication codes used for cohort construction is provided in Supplementary Table S1.

Patients were categorized into two groups based on their pharmaceutical treatment: the finerenone cohort (Cohort A, *n* = 389) and the SGLT2 inhibitor cohort (Cohort B, *n* = 52,242). The index event for each patient was defined as the date of first initiation of the respective study medication (finerenone or an SGLT2 inhibitor) occurring on or after the date the patient met all base population diagnostic criteria for CKD and HF with preserved ejection fraction, thereby establishing a new-user design in which treatment exposure followed confirmed clinical diagnosis. The index date was defined as the first recorded prescription of the study drug (finerenone or SGLT2 inhibitor) following documentation of both CKD and HFpEF diagnoses. A 1-day lag period was applied to minimize the inclusion of events occurring on the same day as treatment initiation. Patients with documented outcomes prior to the start of follow-up were excluded. To ensure a new-user, mutually exclusive cohort design, patients in the finerenone group were required to have no prior or subsequent exposure to SGLT2 inhibitors, and similarly, patients in the SGLT2 inhibitor group could not have any record of finerenone use. Therefore, individuals could not contribute to both cohorts or receive both therapies during follow-up.

To ensure temporal separation between treatment initiation and outcome assessment and to minimize potential reverse causation and misclassification bias, a 1-day lag period was applied, with follow-up for all outcomes beginning exactly one day after the index event. Patients who experienced the outcome of interest prior to the start of the follow-up window were excluded from the respective analyses, ensuring that all evaluated events represented incident outcomes occurring after therapy initiation.

### 2.3. Study Endpoints

The analysis evaluated outcomes over two follow-up periods: 180 days and 365 days. Due to small event counts and data suppression rules, acute kidney injury (AKI) could not be reported for 180 days. The primary endpoints were all-cause mortality and HF hospitalization. Secondary outcomes included all-cause hospitalization or emergency room (ER) visits. Safety outcomes were assessed through the incidence of hyperkalemia (potassium levels ≥ 5.5 mmol/L) and acute kidney injury (AKI). Laboratory trends were also evaluated, specifically focusing on creatinine elevations of ≥2.0 mg/dL.

HF hospitalization was defined as the first inpatient admission with a primary diagnosis of HF, identified using ICD-10-CM code I50. Only encounters classified as inpatient admissions were included. To ensure evaluation of incident events, patients with a history of HF hospitalization prior to the index date were excluded from the respective outcome analysis. Time-to-event analyses were performed using Kaplan–Meier methods and Cox proportional hazards models, with follow-up beginning one day after treatment initiation.

### 2.4. Statistical Analysis

To mitigate baseline differences between the cohorts, PSM was conducted. From a pool of eligible patients (335 in the Finerenone group and 48,296 in the SGLT2 inhibitor group), 1:1 matching was performed, resulting in balanced cohorts of 333 patients each [9]. Matching criteria included demographic factors (age, sex, and race) as well as various comorbid conditions and medications. Although PSM was used to improve covariate balance, it cannot eliminate confounding by indication, as treatment assignment in real-world settings is inherently influenced by clinical severity, physician preference, and other unmeasured factors that may not be fully captured in structured electronic health record data. Despite PSM, residual confounding likely persists, particularly due to imbalance in variables such as [BNP, medications, etc.]. This may bias results in favor of finerenone, as patients in the SGLT2 inhibitor group appeared to have higher baseline disease severity. Covariate balance was assessed using standardized mean differences (SMD), with a threshold of <0.10 indicating adequate balance [10]. Although the number of covariates was relatively high, prior simulation studies have demonstrated that propensity score models remain robust at lower events-per-variable ratios (approximately 5–10), as their primary goal is covariate balance rather than causal inference [11,12]. Although inverse probability of treatment weighting (IPTW) can preserve the full study population, its performance may be compromised in settings with substantial imbalance in treatment group sizes, as extreme weights can introduce instability and increase variance. Given the marked disparity in cohort sizes in the present study, PSM was selected to achieve more stable covariate balance and to facilitate clinically interpretable comparisons between treatment groups. The number of patients included in each outcome analysis varied due to missing laboratory values or incomplete follow-up, which is reflected in the denominators reported for each endpoint. Given the limited number of patients in the finerenone group, 1:1 PSM was selected to optimize covariate balance and maintain interpretability of treatment effect estimates.

**Figure 1:**
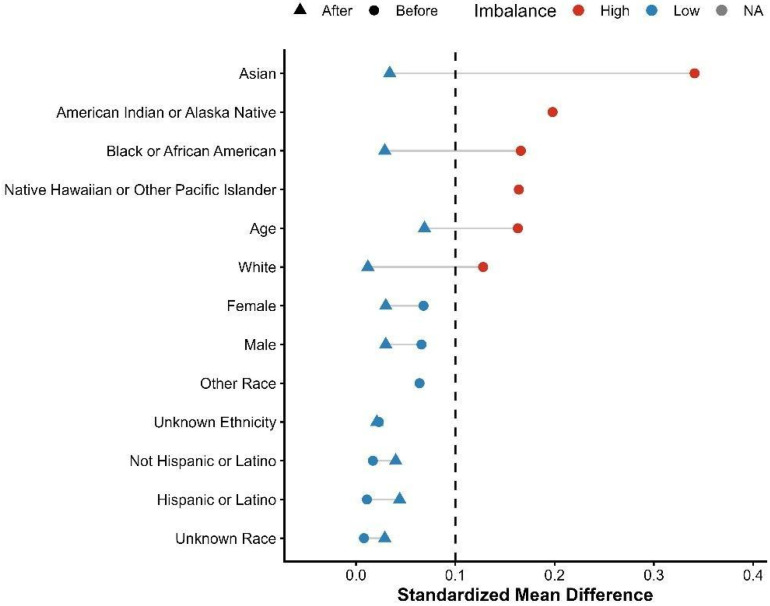
Love plot depicting standardized mean differences (SMDs) for baseline demographic characteristics before and after propensity score matching.

**Figure 2:**
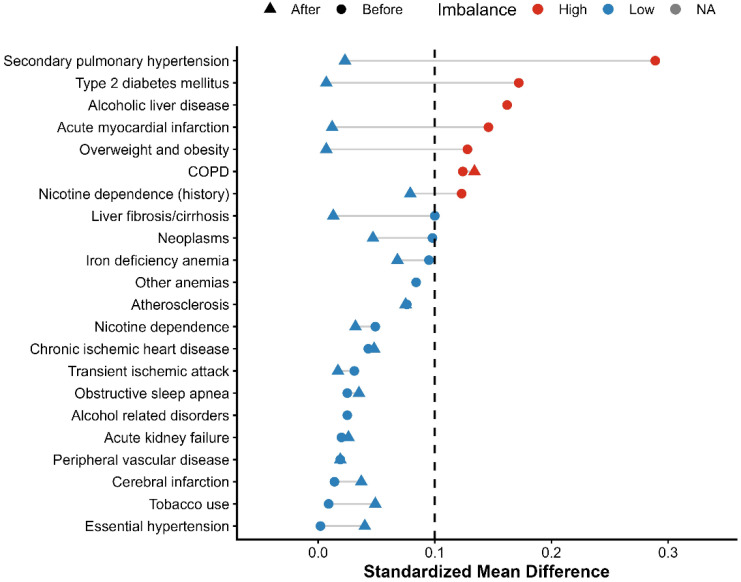
Love plot showing standardized mean differences (SMDs) for baseline comorbidities before and after propensity score matching.

**Figure 3:**
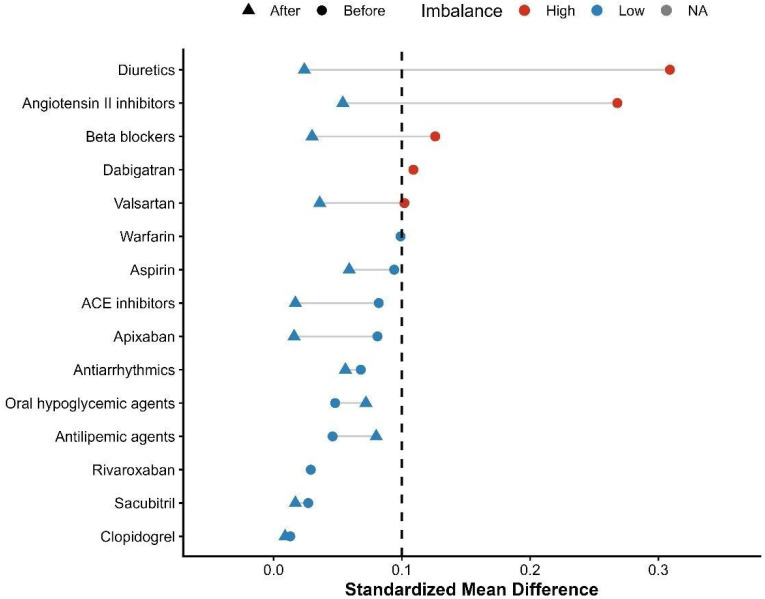
Love plot illustrating standardized mean differences (SMDs) for baseline medication use before and after propensity score matching.

**Figure 4:**
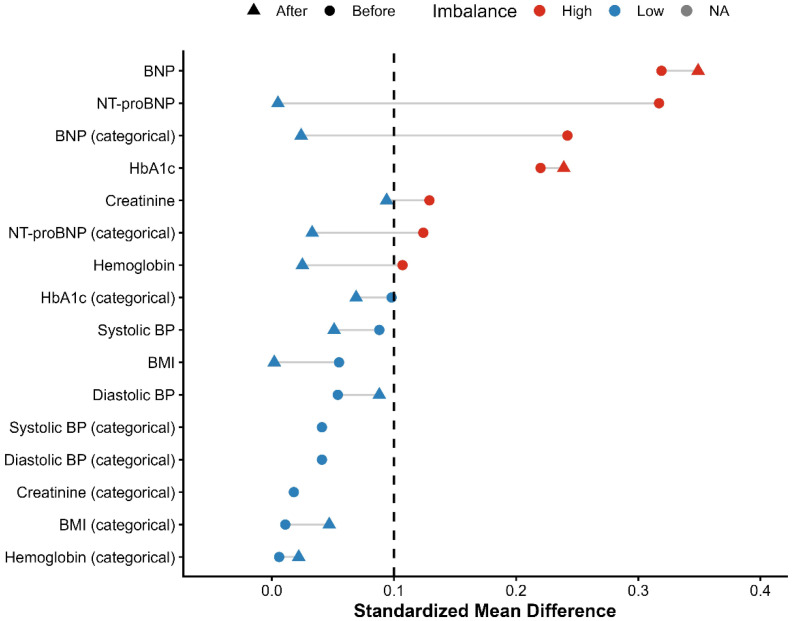
Love plot presenting standardized mean differences (SMDs) for baseline laboratory parameters before and after propensity score matching.

Incidence risks and measures of association specifically Risk Ratios (RRs) and Odds Ratios (ORs) with 95% confidence intervals (CIs)—were calculated through the TriNetX Compare Outcomes model. Time-to-event data were visualized using Kaplan–Meier survival curves, and differences between cohorts were tested with the log-rank test. Hazard ratios (HRs) and 95% CIs were derived from Cox proportional hazards regression models conducted within the TriNetX platform [13,14]. A two-sided *p*-value of less than 0.05 was used to determine statistical significance for all tests. Love plots were generated for demographics, comorbidities, medication use, and laboratory parameters. These are presented in Figure 1, Figure 2, Figure 3 and Figure 4, respectively. The proportional hazards assumption was assessed using the built-in TriNetX proportionality test (based on Schoenfeld residuals), with statistical significance evaluated using chi-square testing. (Supplementary Table S2). No formal adjustment for multiple comparisons was performed.

## 3. Results

### 3.1. Patient Characteristics

**Figure 5:**
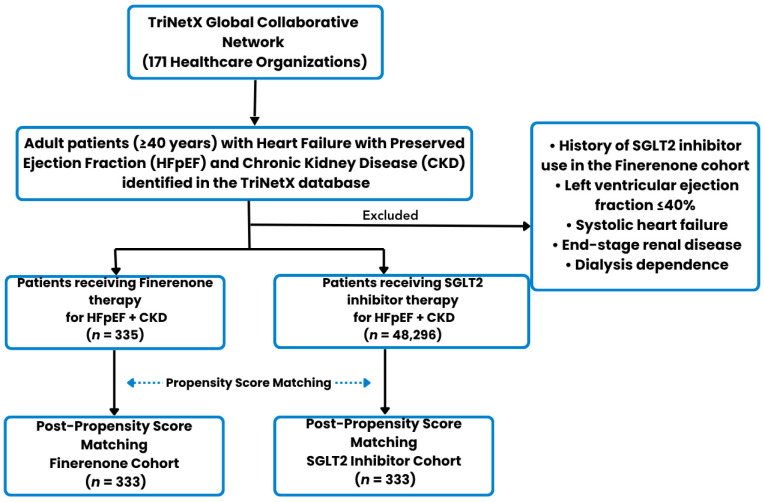
Study Flow Diagram.

A total of 335 patients receiving finerenone and 48,296 patients receiving SGLT2 inhibitor therapy were identified in the initial population (Supplementary Figure S1). After PSM, the cohorts were successfully balanced, resulting in 333 matched patients in each group (Supplementary Figure S1). A detailed study flow diagram outlining cohort selection, inclusion, and exclusion criteria is provided in Figure 5. Although matching improved balance, several covariates remained above the conventional threshold of 0.10, indicating residual imbalance. Baseline demographic and clinical characteristics before and after matching are detailed in Table 1. By design, the two treatment cohorts were mutually exclusive, with no overlap in exposure during follow-up.

Before PSM, the mean age at index was 72.0 ± 10.1 years in the finerenone group and 73.7 ± 10.7 years in the SGLT2 inhibitor group (SMD = 0.163). Regarding sex distribution, females comprised 53.4% of the finerenone group and 50.0% of the SGLT2 inhibitor group (SMD = 0.068), while males accounted for 46.6% and 49.9%, respectively (SMD = 0.066). Racially, White patients represented 57.6% in the finerenone group and 63.9% in the SGLT2 inhibitor group (SMD = 0.128), while Black or African American patients comprised 11.6% and 17.5%, respectively (SMD = 0.166). Ethnically, patients not identified as Hispanic or Latino made up 70.7% of the finerenone group and 70.0% of the SGLT2 inhibitor group (SMD = 0.017), whereas Hispanic or Latino patients represented 4.8% and 4.5%, respectively (SMD = 0.011), with unknown ethnicity reported in 24.5% vs. 25.5% (SMD = 0.023). Baseline comorbidities included essential hypertension in 72.5% of the finerenone group and 72.6% of the SGLT2 inhibitor group (SMD = 0.002), type 2 diabetes mellitus in 71.0% vs. 63.0% (SMD = 0.172), and overweight/obesity in 30.1% vs. 36.2% (SMD = 0.128). Acute kidney injury was reported in 30.4% of the finerenone group and 29.5% of the SGLT2 inhibitor group (SMD = 0.020), chronic ischemic heart disease affected 42.7% vs. 44.8% (SMD = 0.043), and cerebral infarction occurred in 6.9% vs. 6.5% (SMD = 0.014). Atherosclerosis was observed in 14.0% vs. 11.5% (SMD = 0.076). Regarding pharmacological profiles, SGLT2 inhibitor patients had higher use of diuretics (49.6% vs. 64.7%; SMD = 0.309) and beta-blockers (51.9% vs. 58.2%; SMD = 0.126). The finerenone group showed higher use of Angiotensin II inhibitors (44.2% vs. 31.3%; SMD = 0.268) and valsartan (12.5% vs. 9.4%; SMD = 0.102). Antithrombotic and anticoagulant use was similar, including aspirin (29.3% vs. 33.6%; SMD = 0.094), apixaban (17.3% vs. 20.5%; SMD = 0.081), and clopidogrel (12.2% vs. 12.7%; SMD = 0.013). ACE inhibitors (15.5% vs. 18.6%; SMD = 0.082), antilipemic agents (58.2% vs. 60.4%; SMD = 0.046), and oral hypoglycemic agents (24.2% vs. 26.3%; SMD = 0.048) were balanced. Anti-arrhythmic use was also comparable: 37.6% in the finerenone group vs. 40.9% in the SGLT2 inhibitor group (SMD = 0.068). Before PSM, baseline laboratory values were similar between the two groups. The mean hemoglobin level was 12.1 ± 2.3 g/dL in the finerenone group and 11.8 ± 2.3 g/dL in the SGLT2 inhibitor group (SMD = 0.107). The mean BMI was 32.7 ± 8.9 kg/m^2^ in the finerenone group and 33.2 ± 8.5 kg/m^2^ in the SGLT2 inhibitor group (SMD = 0.055). Serum creatinine was 1.6 ± 0.6 mg/dL in the finerenone group and 2.7 ± 12.0 mg/dL in the SGLT2 inhibitor group. Blood pressure values were similar: mean systolic/diastolic BP was 133.2 ± 22.1/70.2 ± 12.6 mmHg in the finerenone group and 131.3 ± 21.5/70.9 ± 13.4 mmHg in the SGLT2 inhibitor group. BNP was 292.0 ± 396.7 pg/mL vs. 880.0 ± 2580.4 pg/mL (SMD = 0.319), NT-proBNP was 2144.0 ± 2645.1 pg/mL vs. 3642.3 ± 6140.2 pg/mL (SMD = 0.317), and HbA1c was 6.9 ± 1.3% vs. 7.2 ± 1.7% (SMD = 0.220).

Following PSM, the study included 666 patients (333 per group), achieving a high degree of balance across baseline variables (all standardized mean differences [SMD] < 0.15) (Table 1). The average age at index was 72.4 years (SMD = 0.069). Females comprised 52.5% of the population (SMD = 0.030). The racial distribution included 58.3% White patients (SMD = 0.012) and 11.3% Black or African American patients (SMD = 0.029). Essential hypertension was present in 71.8% of patients (SMD = 0.040), and 71.4% had type 2 diabetes mellitus (SMD = 0.007). Chronic ischemic heart disease and acute kidney injury were observed in 43.5% (SMD = 0.048) and 30.6% (SMD = 0.026) of the patients, respectively. Overweight or obesity affected 29.9% of the cohort (SMD = 0.007), while 27.8% had neoplasms (SMD = 0.047) and 27.3% had other anemias (SMD < 0.001).

The proportional hazards assumption was satisfied for all primary outcomes (Supplementary Table S2).

### 3.2. Primary Outcomes

#### 3.2.1. All-Cause Mortality

**Figure 6:**
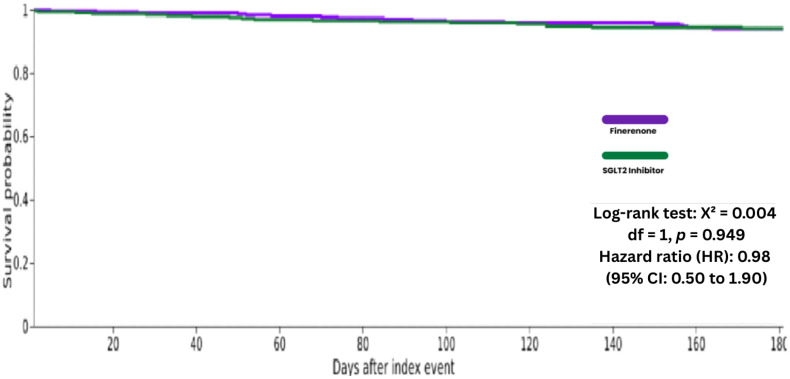
Kaplan–Meier curves for All-Cause Mortality at 6 Months Follow-up.

At the 6 months follow-up, 17 of 332 patients (5.1%) in the finerenone group and 18 of 332 patients (5.4%) in the SGLT2 inhibitor group experienced all-cause mortality outcome. Kaplan–Meier analysis found no difference in hazard between groups at 6 months (HR 0.98; 95% CI 0.50 to 1.90; log-rank *p* = 0.949) (Table 2 and Figure 6).

**Figure 7:**
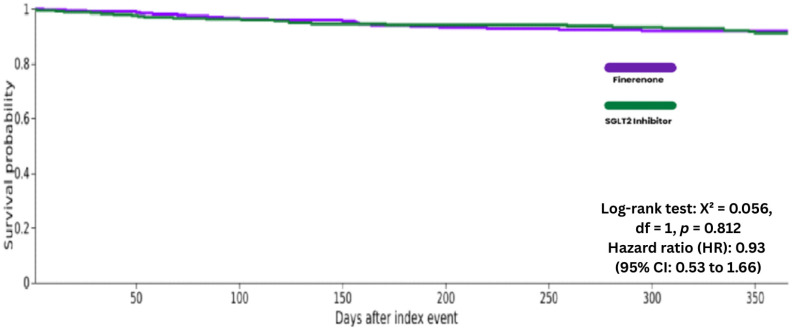
Kaplan–Meier curves for All-Cause Mortality at 1 Year Follow-up.

At 1 year follow-up, all-cause mortality was observed in 22 of 332 patients (6.6%) in the finerenone group and 25 of 332 patients (7.5%) in the SGLT2 inhibitor group. Kaplan–Meier survival analysis showed an HR of 0.93 (95% CI: 0.53 to 1.66; log-rank *p* = 0.812), suggesting that the hazard of mortality was similar between the two groups over 1 year (Table 2 and Figure 7).

#### 3.2.2. HF Hospitalization

**Figure 8:**
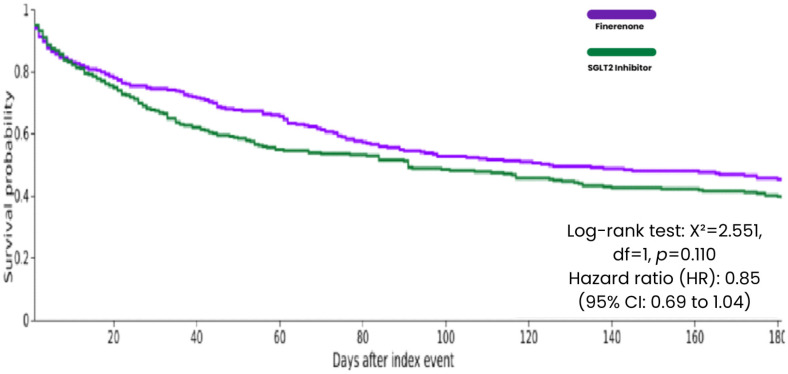
Kaplan–Meier curves for Heart Failure Hospitalization at 6 Months Follow-up.

At the 6 months follow-up, heart failure (HF) hospitalization occurred in 168 of 333 patients (50.4%) in the finerenone group and 190 of 333 patients (57.1%) in the SGLT2 inhibitor group. Kaplan–Meier time-to-event analysis showed a non-significant reduction in HF hospitalization with finerenone (HR 0.85; 95% CI: 0.69 to 1.04; log-rank *p* = 0.110). Although the point estimate favored finerenone, the result did not reach statistical significance and should be interpreted as a hypothesis-generating finding requiring further confirmation (Table 2 and Figure 8).

**Figure 9:**
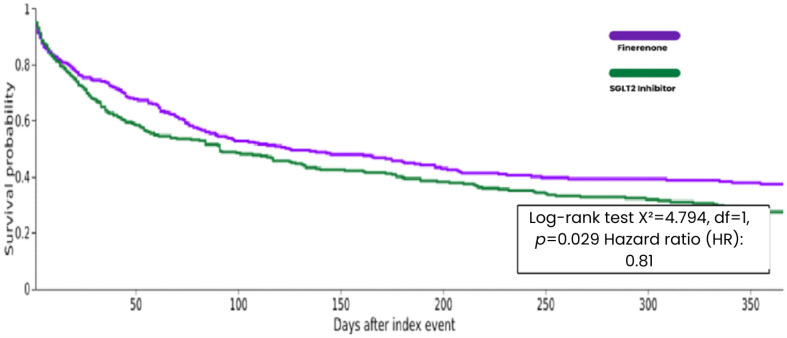
Kaplan–Meier curves for Heart Failure Hospitalization at 1 Year Follow-up.

At 1 year follow-up, HF hospitalization was reported in 187 of 333 patients (56.2%) in the finerenone group and 220 of 333 patients (66.1%) in the SGLT2 inhibitor group. Kaplan–Meier analysis revealed a nominally significant lower risk of HF hospitalization with finerenone therapy at 1 year (HR 0.81; 95% CI: 0.66 to 0.99; log-rank *p* = 0.029), which was not robust after adjustment for multiple comparisons (Table 2 and Figure 9).

To assess the robustness of the observed association to unmeasured confounding, the E-value for the hazard ratio of 0.81 was calculated to be approximately 1.77, while the E-value for the upper confidence bound (0.99) was 1.11. This suggests that a relatively modest unmeasured confounder could potentially explain the observed association.

### 3.3. Secondary Outcomes

#### 3.3.1. All-Cause Hospitalization/ER Visits

At the 6 months follow-up, all-cause hospitalization or ER visits were reported in 135 of 333 patients (40.5%) receiving finerenone and in 133 of 333 patients (39.9%) treated with an SGLT2 inhibitor. Kaplan–Meier time-to-event analysis found no statistically significant difference between the groups at 6 months (HR 1.07; 95% CI 0.84 to 1.36; log-rank *p* = 0.600) (Table 2 and Supplementary Figure S2).

At 1 year follow-up, all-cause hospitalizations were observed in 160 of 333 patients (48.1%) in the finerenone group and 163 of 333 patients (48.9%) in the SGLT2 inhibitor group. Kaplan–Meier analysis revealed an HR of 1.04 (95% CI: 0.83 to 1.29; log-rank *p* = 0.760), suggesting no significant difference in risk at 1 year (Table 2 and Supplementary Figure S2).

#### 3.3.2. Hyperkalemia ≥ 5.5 mmol/L

At the 6 months follow-up, hyperkalemia ≥ 5.5 mmol/L was observed in 13 of 252 patients (5.2%) receiving finerenone and in 14 of 257 patients (5.5%) treated with an SGLT2 inhibitor. There was no significant difference between groups at 6 months based on Kaplan–Meier time-to-event analysis (HR 0.98; 95% CI 0.46 to 2.09; log-rank *p* = 0.961) (Table 2 and Supplementary Figure S3).

At 1 year, hyperkalemia ≥5.5 mmol/L was reported in 19 of 252 patients (7.5%) in the finerenone group compared with 18 of 257 patients (7.0%) in the SGLT2 inhibitor group. Kaplan–Meier analysis yielded an HR of 1.14 (95% CI: 0.60 to 2.17; log-rank *p* = 0.691), demonstrating a non-significant trend toward higher risk with finerenone at 1 year follow-up (Table 2 and Supplementary Figure S3).

#### 3.3.3. Creatinine ≥ 2.0

At the 6 months follow-up, creatinine ≥ 2.0 was reported in 14 of 176 patients (8.0%) receiving finerenone and in 26 of 207 patients (12.6%) treated with an SGLT2 inhibitor. Kaplan–Meier time-to-event analysis yielded an HR of 0.63 (95% CI: 0.33 to 1.21; log-rank *p* = 0.162), suggesting a non-significant trend toward lower hazard with finerenone at 6 months (Table 2 and Supplementary Figure S4).

At 1 year, creatinine ≥2.0 was observed in 25 of 176 patients in the finerenone group compared with 30 of 207 patients in the SGLT2 inhibitor group. Kaplan–Meier analysis produced an HR of 0.99 (95% CI: 0.58 to 1.68; log-rank *p* = 0.964), suggesting no significant difference with finerenone therapy during the 1-year follow-up period (Table 2 and Supplementary Figure S4).

#### 3.3.4. Acute Kidney Injury (AKI)

6 months AKI outcome was not available due to insufficient event counts under TriNetX reporting thresholds.

At 1 year, AKI was reported in 12 of 144 patients (8.3%) receiving finerenone and in 20 of 158 patients (12.7%) treated with an SGLT2 inhibitor. Kaplan–Meier time-to-event analysis yielded an HR of 0.68 (95% CI: 0.33–1.38; log-rank *p* = 0.281), suggesting a non-significant trend toward lower hazard of AKI with finerenone therapy at 1 year (Table 2 and Supplementary Figure S5).

## 4. Discussion

**Figure 10:**
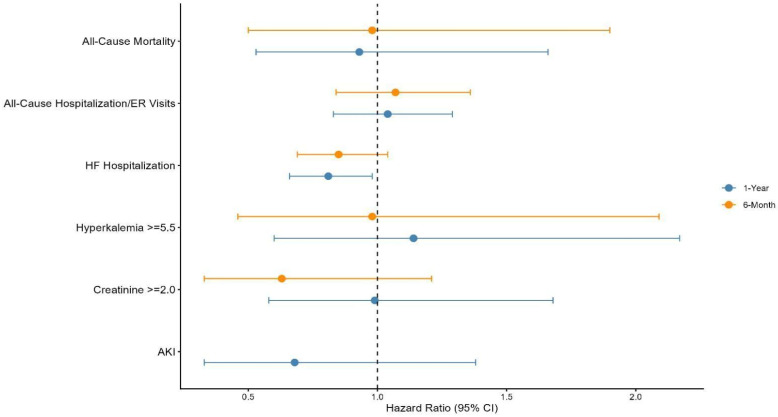
Forest plot of clinical outcomes comparing Finerenone versus SGLT2 inhibitor therapy in patients with HFpEF and CKD. Acute kidney injury (AKI) at 6 months was not reported because the event count in one cohort was <10. In accordance with TriNetX Research Network privacy policies and Health Insurance Portability and Accountability Act (HIPAA) compliance, outcomes with counts <10 are suppressed to protect patient confidentiality.

After PSM, 333 patients were included in each group, achieving a high degree of balance across baseline demographic and clinical characteristics between the finerenone and SGLT2 inhibitor groups. Overall, all-cause mortality and all-cause hospitalization or emergency department visits were similar between finerenone and SGLT2 inhibitors at both 6 months and 1 year, suggesting largely neutral effects on these broad outcomes. In contrast, finerenone was associated with a nominally significant lower risk of HF hospitalization at 1-year follow-up, which should be interpreted cautiously given its hypothesis-generating nature; however, no significant differences were observed at 6 months. Regarding safety profiles, the incidence of hyperkalemia (≥5.5 mmol/L) and elevated creatinine (≥2.0 mg/dL) was comparable between the two groups at both follow-up intervals. Notably, the incidence of acute kidney injury was lower with finerenone, but this difference was not statistically significant, suggesting a similar renal profile to SGLT2 inhibitors (Figure 10) (Graphical Abstract). Residual imbalance may persist despite matching, even when SMD thresholds are achieved [10].

The favorable cardiovascular and renal outcomes observed with finerenone in our study may reflect the complex interplay between mineralocorticoid receptor (MR) overactivation, inflammation, and fibrotic remodeling in patients with CKD and type 2 diabetes. In populations with a high burden of comorbidities, including essential hypertension and HF, persistent MR signaling contributes to a proinflammatory and profibrotic milieu that enhances organ damage. Targeting this pathway by finerenone, a non-steroidal mineralocorticoid receptor antagonist, provides potent protection against cardiorenal progression [15,16]. The ability of finerenone to block mineralocorticoid receptors without significant changes in hemodynamic parameters may help preserve cardiac function and could contribute to the lower HF hospitalization observed at 1 year, though causality cannot be established [17,18].

Finerenone selectively binds to the mineralocorticoid receptor, preventing the recruitment of transcriptional co-activators involved in the expression of proinflammatory and profibrotic genes [19]. Apart from its anti-fibrotic ability, experimental and clinical evidence suggests that this agent can influence vascular and renal biology through the modulation of oxidative stress and endothelial function [20]. Thus, by specifically inhibiting MR-mediated damage, with similar rates of hyperkalemia and creatinine elevation compared with SGLT2 inhibitors in this study, finerenone may provide a more stable long-term protective strategy. In contrast, while SGLT2 inhibitors offer robust benefits [21], the incidence of acute kidney injury was lower with finerenone, but this difference was not statistically significant, suggesting a similar renal profile compared with SGLT2 inhibitors in this specific patient population.

The observed reduction in HF hospitalization at 1 year was modest, borderline in statistical significance, and not consistent across earlier timepoints, nor accompanied by a mortality benefit. Therefore, this finding should be considered hypothesis-generating rather than confirmatory. In prior large randomized controlled trials (RCTs) evaluating finerenone in patients with CKD and type 2 diabetes, the drug consistently resulted in reduction in composite cardiovascular outcomes primarily due to its effect on the reduction in heart failure-related events. In the large sample-based FIGARO-DKD trial by Pitt et al., showed that finerenone significantly reduced cardiovascular death, non-fatal myocardial infarction, non-fatal stroke, or hospitalization for HF compared with placebo, with HF hospitalization proved to be as a major contributor to this benefit, while all-cause mortality was similar between the groups [22]. When pooled with FIDELIO-DKD data in the FIDELITY analysis by Agarwal et al., finerenone showed an overall cardiovascular risk reduction and kidney-related outcomes in patients with CKD and type 2 diabetes mellitus [15]. In contrast, large SGLT2 inhibitor outcome trials such as DAPA-HF by John. J.V et al., and EMPEROR by Anker et al., have revealed robust reductions in composite endpoints including HF hospitalizations and cardiovascular death, with consistent mortality benefits for SGLT2 inhibitors compared with placebo across patients with reduced ejection fraction and with preserved ejection fraction respectively [23,24]. Our real-world analysis complements these RCT results, showing neutral all-cause mortality and a nominal reduction in HF hospitalization at one year, suggesting a potential differential effect that warrants further investigation. Notably, SGLT2 inhibitors have consistently confirmed reductions in HF hospitalization and cardiovascular mortality across multiple RCTs, which contrasts with the neutral mortality findings observed in this real-world analysis. The relatively high rates of HF hospitalization observed in this study warrant careful interpretation. These elevated event rates may reflect differences in coding practices within administrative databases, inclusion of a higher-risk population with multiple comorbidities, or characteristics specific to the TriNetX network, such as capture of hospital-based encounters. Additionally, variations in healthcare utilization patterns and coding intensity across participating institutions may contribute to increased event ascertainment. These factors may limit the direct comparability of event rates with those reported in clinical trials or other real-world datasets.

Additionally, SGLT2 inhibitors have emerged as pleiotropic agents with cardiovascular, renal, and systemic metabolic benefits beyond glycemic control. Their effects extend beyond natriuresis and osmotic diuresis to include improvement in myocardial energy efficiency via ketone utilization, reduction in sympathetic activation, and attenuation of inflammatory and oxidative stress pathways. Additional benefits include favorable modulation of vascular function, endothelial health, and metabolic parameters such as uric acid and hepatic fat content. Mechanistically, these agents are linked to improved cellular energy signaling, autophagy, and mitochondrial function, which together contribute to cardiovascular and renal protection. These pleiotropic effects may provide a biological basis for the consistent reductions in heart failure hospitalization and cardiovascular outcomes observed in clinical trials. This mechanistic framework has been well described in contemporary reviews by Theophilis et al. and Packer et al. [25,26]. Similarly, a state-of-the art review by Zelniker et al. also demonstrated reductions in cardiovascular and renal events across SGLT2 inhibitors [27].

Importantly, while direct head-to-head comparisons between finerenone and SGLT2 inhibitors have not been conducted in large RCTs, a meta-analysis of SGLT2 inhibitor trials by Cardoso et al., have consistently reported significant reductions in all-cause and cardiovascular mortality as well as HF hospitalization as compared with placebo, across HF populations [28]. SGLT2 inhibitors are recommended as foundational therapies in contemporary HF and CKD guidelines because they significantly reduce cardiovascular death, hospitalization for HF, and kidney disease progression, and carry Class I recommendations for patients with type 2 diabetes and symptomatic HF [29]. By contrast, finerenone’s major RCT benefits have been most evident in HF and cardiovascular event reduction in CKD patients, with mortality effects more modest or inconsistent [30]. Therefore, our finding of a nominally statistically significant lower hazard of HF hospitalization with finerenone in real-world matched cohorts provides real-world context to existing RCT evidence and highlights a potential difference in observed outcomes that warrants further investigation in prospective comparative studies.

### Limitations

Several limitations of this study warrant consideration. First, the retrospective, observational design utilizing the TriNetX federated database carries inherent risks of selection bias and unmeasured confounding that PSM cannot fully eliminate. Notably, there was a massive imbalance in the pre-matched groups (335 vs. 48,296), consistent with strong treatment selection bias and confounding by indication; propensity-score matching may not fully account for these factors. Despite the use of PSM, residual confounding by indication remains a key limitation. Treatment allocation in routine clinical practice is influenced by disease severity, comorbidity burden, and physician decision-making, which may not be fully captured in the available variables. Second, the reliance on ICD-10-CM and CPT codes for cohort definition and outcome adjudication introduces potential misclassification bias, as administrative codes may not fully capture clinical complexity or the precise timing of events. In particular, the definition of HFpEF relied on ICD-based diagnostic coding and exclusion of patients with LVEF ≤ 40%, without direct echocardiographic confirmation. This approach introduces a significant risk of misclassification bias, especially in differentiating HFpEF from HFmrEF or patients with recovered ejection fraction. Given the dynamic nature of left ventricular function and the limitations of administrative coding, the study cohort may include a heterogeneous mix of HF phenotypes. Such misclassification could bias results toward the null or obscure true treatment effects, limiting the precision of the comparative effectiveness estimates. Third, the database captures prescribing patterns rather than actual medication adherence; we could not verify whether patients remained compliant with their assigned therapy or whether dose titrations occurred. Fourth, despite unit harmonization within the platform, missingness in granular clinical data—such as baseline albuminuria levels or specific HF phenotypes—precluded more detailed subgroup analyses of treatment efficacy. Multiple outcomes were evaluated without formal adjustment for multiplicity. Applying a Bonferroni correction (α = 0.05/6 = 0.0083), the observed association for HF hospitalization at 1 year (*p* = 0.029) would not meet the adjusted threshold for statistical significance. Therefore, this finding should be interpreted as exploratory. Residual confounding due to unavailable variables such as albuminuria, HF severity, and echocardiographic parameters remains a major limitation. Moreover, the relatively high observed rates of HF hospitalization may reflect differences in coding practices, inclusion of high-risk populations, or potential misclassification within administrative data, and should be interpreted cautiously. Formal sensitivity analyses were not performed, and model calibration metrics (e.g., C-statistics or calibration plots) were not available within the TriNetX platform, which may limit the robustness of the findings. Furthermore, baseline creatinine values showed variability across patients, likely reflecting outliers and heterogeneity in the dataset; therefore, these values should be interpreted cautiously. The matched sample included only 333 patients per group, limiting statistical power and generalizability; many estimates, particularly safety outcomes, have wide confidence intervals and should be interpreted cautiously. The relatively small sample size following matching reflects the limited number of finerenone users available in the database rather than the matching strategy itself. Alternative approaches such as inverse probability weighting were not performed and may be considered in future analyses. Finally, the mutually exclusive cohort design, while ensuring treatment separation, may introduce selection bias by excluding patients who switched or received combination therapy, potentially limiting generalizability. Additionally, exposure was based on prescription records in TriNetX, which do not capture adherence, persistence, or dose titration; thus, actual medication use over time cannot be fully verified.

## 5. Conclusions

In this real-world comparative analysis of patients with HFpEF and CKD, treatment with finerenone was associated with similar risks of all-cause mortality, overall hospitalization, and renal safety outcomes compared with SGLT2 inhibitors during follow-up. Finerenone showed a modest, borderline reduction in HF hospitalization at 1 year, without consistency or mortality benefit, and should be considered hypothesis-generating. These findings suggest a potential comparable cardiorenal therapeutic profile for finerenone in this high-risk population, with possible benefits in reducing heart failure–related events. Prospective head-to-head randomized trials are warranted to further clarify the optimal sequencing and integration of these therapies in patients with HFpEF and CKD.

## Figures and Tables

**Table 1:** Baseline characteristics of finerenone and SGLT2 inhibitor cohorts before and after propensity score matching.

Characteristic	Finerenone Group (*n* = 335)	SGLT2 Inhibitor Group (*n* = 48,296)	Std. Diff.	Finerenone Group (*n* = 333)	SGLT2 Inhibitor Group (*n* = 333)	Std. Diff.
	Before PSM			After PSM		
Demographics						
Age at Index (years), mean ± SD	72.0 ± 10.1	73.7 ± 10.7	0.163	72.0 ± 10.1	72.8 ± 11.1	0.069
Female	179 (53.4%)	24,165 (50.0%)	0.068	177 (53.2%)	172 (51.7%)	0.03
Male	156 (46.6%)	24,085 (49.9%)	0.066	156 (46.8%)	161 (48.3%)	0.03
Black or African American	39 (11.6%)	8433 (17.5%)	0.166	39 (11.7%)	36 (10.8%)	0.029
White	193 (57.6%)	30,841 (63.9%)	0.128	193 (58.0%)	195 (58.6%)	0.012
American Indian or Alaska Native	10 (3.0%)	209 (0.4%)	0.198	10 (3.0%)	10 (3.0%)	<0.001
Native Hawaiian or Other Pacific Islander	10 (3.0%)	372 (0.8%)	0.164	10 (3.0%)	10 (3.0%)	<0.001
Asian	52 (15.5%)	2543 (5.3%)	0.341	51 (15.3%)	47 (14.1%)	0.034
Other Race	10 (3.0%)	963 (2.0%)	0.064	10 (3.0%)	10 (3.0%)	<0.001
Unknown Race	35 (10.4%)	4935 (10.2%)	0.008	35 (10.5%)	38 (11.4%)	0.029
Unknown Ethnicity	82 (24.5%)	12,312 (25.5%)	0.023	82 (24.6%)	79 (23.7%)	0.021
Not Hispanic or Latino	237 (70.7%)	33,792 (70.0%)	0.017	235 (70.6%)	241 (72.4%)	0.04
Hispanic or Latino	16 (4.8%)	2192 (4.5%)	0.011	16 (4.8%)	13 (3.9%)	0.044
***Comorbidities***						
Essential (primary) hypertension	243 (72.5%)	35,081 (72.6%)	0.002	242 (72.7%)	236 (70.9%)	0.04
Type 2 diabetes mellitus	238 (71.0%)	30,420 (63.0%)	0.172	237 (71.2%)	238 (71.5%)	0.007
Overweight and obesity	101 (30.1%)	17,466 (36.2%)	0.128	100 (30.0%)	99 (29.7%)	0.007
Acute kidney failure	102 (30.4%)	14,269 (29.5%)	0.02	100 (30.0%)	104 (31.2%)	0.026
Chronic ischemic heart disease	143 (42.7%)	21,657 (44.8%)	0.043	141 (42.3%)	149 (44.7%)	0.048
Acute myocardial infarction	20 (6.0%)	4780 (9.9%)	0.146	20 (6.0%)	21 (6.3%)	0.012
Cerebral infarction	23 (6.9%)	3143 (6.5%)	0.014	23 (6.9%)	20 (6.0%)	0.037
Transient cerebral ischemic attacks and related syndromes	10 (3.0%)	1201 (2.5%)	0.031	10 (3.0%)	11 (3.3%)	0.017
Atherosclerosis	47 (14.0%)	5553 (11.5%)	0.076	47 (14.1%)	56 (16.8%)	0.075
Peripheral vascular disease, unspecified	37 (11.0%)	5045 (10.4%)	0.019	37 (11.1%)	39 (11.7%)	0.019
Other chronic obstructive pulmonary disease	60 (17.9%)	11,061 (22.9%)	0.124	59 (17.7%)	77 (23.1%)	0.134
Obstructive sleep apnea	81 (24.2%)	12,208 (25.3%)	0.025	81 (24.3%)	86 (25.8%)	0.035
Iron deficiency anemia	40 (11.9%)	7339 (15.2%)	0.095	39 (11.7%)	32 (9.6%)	0.068
Other anemias	92 (27.5%)	11,498 (23.8%)	0.084	91 (27.3%)	91 (27.3%)	<0.001
Fibrosis and cirrhosis of liver	19 (5.7%)	1728 (3.6%)	0.1	19 (5.7%)	18 (5.4%)	0.013
Alcoholic liver disease	10 (3.0%)	379 (0.8%)	0.162	10 (3.0%)	10 (3.0%)	<0.001
Neoplasms	97 (29.0%)	11,884 (24.6%)	0.098	96 (28.8%)	89 (26.7%)	0.047
Tobacco use	10 (3.0%)	1515 (3.1%)	0.009	10 (3.0%)	13 (3.9%)	0.049
Personal history of nicotine dependence	64 (19.1%)	11,673 (24.2%)	0.123	64 (19.2%)	54 (16.2%)	0.079
Alcohol-related disorders	10 (3.0%)	1243 (2.6%)	0.025	10 (3.0%)	10 (3.0%)	<0.001
Nicotine dependence	27 (8.1%)	4559 (9.4%)	0.049	27 (8.1%)	30 (9.0%)	0.032
Other secondary pulmonary hypertension	24 (7.2%)	7915 (16.4%)	0.289	24 (7.2%)	26 (7.8%)	0.023
***Medications***						
Beta blockers	174 (51.9%)	28,114 (58.2%)	0.126	174 (52.3%)	179 (53.8%)	0.03
Diuretics	166 (49.6%)	31,241 (64.7%)	0.309	166 (49.8%)	170 (51.1%)	0.024
Antiarrhythmics	126 (37.6%)	19,775 (40.9%)	0.068	125 (37.5%)	116 (34.8%)	0.056
ACE inhibitors	52 (15.5%)	8991 (18.6%)	0.082	51 (15.3%)	53 (15.9%)	0.017
Warfarin	10 (3.0%)	2369 (4.9%)	0.099	10 (3.0%)	10 (3.0%)	<0.001
Rivaroxaban	15 (4.5%)	2461 (5.1%)	0.029	15 (4.5%)	15 (4.5%)	<0.001
Apixaban	58 (17.3%)	9898 (20.5%)	0.081	58 (17.4%)	60 (18.0%)	0.016
Dabigatran etexilate	0 (0%)	283 (0.6%)	0.109	0 (0%)	0 (0%)	—
Aspirin	98 (29.3%)	16,237 (33.6%)	0.094	97 (29.1%)	106 (31.8%)	0.059
Clopidogrel	41 (12.2%)	6122 (12.7%)	0.013	40 (12.0%)	39 (11.7%)	0.009
Angiotensin II inhibitors	148 (44.2%)	15,112 (31.3%)	0.268	146 (43.8%)	155 (46.5%)	0.054
Valsartan	42 (12.5%)	4518 (9.4%)	0.102	40 (12.0%)	44 (13.2%)	0.036
Sacubitril	11 (3.3%)	1828 (3.8%)	0.027	11 (3.3%)	10 (3.0%)	0.017
Antilipemic agents	195 (58.2%)	29,194 (60.4%)	0.046	193 (58.0%)	206 (61.9%)	0.08
Oral hypoglycemic agents	81 (24.2%)	12,683 (26.3%)	0.048	81 (24.3%)	71 (21.3%)	0.072
***Laboratory Values***						
Hemoglobin (g/dL)	12.1 ± 2.3	11.8 ± 2.3	0.107	12.1 ± 2.3	12.2 ± 2.3	0.025
Hemoglobin 0–0 g/dL	266 (79.4%)	38,473 (79.7%)	0.006	264 (79.3%)	261 (78.4%)	0.022
Natriuretic peptide B (pg/mL)	292.0 ± 396.7	880.0 ± 2580.4	0.319	292.0 ± 396.7	1580.5 ± 5205.7	0.349
Natriuretic peptide B 0–0 pg/mL	55 (16.4%)	12,676 (26.2%)	0.242	55 (16.5%)	58 (17.4%)	0.024
Natriuretic peptide B prohormone N-terminal (pg/mL)	2144.0 ± 2645.1	3642.3 ± 6140.2	0.317	2144.0 ± 2645.1	2131.4 ± 2834.4	0.005
Natriuretic peptide B prohormone N-terminal 0–0 pg/mL	55 (16.4%)	10,273 (21.3%)	0.124	55 (16.5%)	51 (15.3%)	0.033
Hemoglobin A1c (%)	6.9 ± 1.3	7.2 ± 1.7	0.22	6.9 ± 1.3	7.2 ± 1.5	0.239
Hemoglobin A1c 0–0%	213 (63.6%)	28,400 (58.8%)	0.098	212 (63.7%)	223 (67.0%)	0.069
BMI (kg/m^2^)	32.7 ± 8.9	33.2 ± 8.5	0.055	32.7 ± 8.9	32.8 ± 8.7	0.002
BMI 0–0 kg/m^2^	246 (73.4%)	35,235 (73.0%)	0.011	244 (73.3%)	237 (71.2%)	0.047
Blood Pressure, Systolic (mmHg)	133.2 ± 22.1	131.3 ± 21.5	0.088	133.1 ± 22.1	132.1 ± 19.9	0.051
Blood Pressure, Systolic 0–0 mmHg	268 (80.0%)	39,420 (81.6%)	0.041	266 (79.9%)	266 (79.9%)	<0.001
Blood Pressure, Diastolic (mmHg)	70.2 ± 12.6	70.9 ± 13.4	0.054	70.2 ± 12.7	71.4 ± 13.4	0.088
Blood Pressure, Diastolic 0–0 mmHg	268 (80.0%)	39,414 (81.6%)	0.041	266 (79.9%)	266 (79.9%)	<0.001
Creatinine (mg/dL)	1.6 ± 0.6	2.7 ± 12.0	0.129	1.6 ± 0.6	2.3 ± 11.6	0.094
Creatinine 0–0 mg/dL	276 (82.4%)	40,117 (83.1%)	0.018	274 (82.3%)	274 (82.3%)	<0.001

**Table 2:** 6 months and 1 year Clinical Outcomes After Propensity-Score-Matching. HR = Hazard Ratio; CI = Confidence Interval; *n* = population with outcome; N = total population of cohort. Acute kidney injury (AKI) at 6 months was not reported because the event count in one cohort was <10. In accordance with TriNetX Research Network privacy policies and Health Insurance Portability and Accountability Act (HIPAA) compliance, outcomes with counts <10 are suppressed to protect patient confidentiality.

Outcome	6 Months Finerenone (*n*/N)	6 Months SGLT2 Inhibitor (*n*/N)	HR (95% CI)	Log-Rank *p*-Value	1 Year Finerenone (*n*/N)	1 Year SGLT2 Inhibitor (*n*/N)	HR (95% CI)	Log-Rank *p*-Value
**All-Cause Mortality**	17/332	18/332	0.98 (0.50–1.90)	0.949	22/332	25/332	0.93 (0.53–1.66)	0.812
**All-Cause Hospitalization/ER Visits**	135/333	135/333	1.07 (0.84–1.36)	0.6	160/333	163/333	1.04 (0.83–1.29)	0.76
**HF Hospitalization**	168/333	190/333	0.85 (0.69–1.04)	0.11	187/333	220/333	0.81 (0.66–0.99)	0.029
**Hyperkalemia ≥ 5.5 mmol/L**	13/252	14/257	0.98 (0.46–2.09)	0.961	19/252	18/257	1.14 (0.60–2.17)	0.691
**Creatinine ≥ 2.0**	14/176	26/207	0.63 (0.33–1.21)	0.162	25/176	30/207	0.99 (0.58–1.68)	0.964
**AKI**	—	—	—	—	12/144	20/158	0.68 (0.33–1.38)	0.281

## Data Availability

The data that support the findings of this study are available from the TriNetX Research Network. Due to licensing restrictions and data use agreements, the raw data are not publicly available. Access to the TriNetX platform can be obtained through institutional subscription. Aggregate data supporting the findings of this study may be available from the corresponding author upon request and with permission from TriNetX.

## Supplementary Materials

The following supporting information can be downloaded at: https://www.mdpi.com/article/10.3390/biomedicines14051108/s1, Figure S1: Propensity Score Matching; Figure S2: Kaplan–Meier curves for All-Cause Hospitalization/ER Visits at 6-Months and 1-Year Follow-up; Figure S3: Kaplan–Meier curves for Hyperkalemia ≥ 5.5 at 6-Months and 1-Year Follow-up; Figure S4: Kaplan–Meier curves for Creatinine ≥ 2.0 at 6-Months and 1-Year Follow-up; Figure S5: Kaplan–Meier curves for Acute Kidney Injury (AKI) at 1-Year Follow-up; Table S1: Codes and definitions used for cohort selection, exposures, and outcomes; Table S2: Baseline characteristics of finerenone and SGLT2 inhibitor cohorts before and after propensity score matching.

## Abbreviations

HFpEF	Heart Failure with Preserved Ejection Fraction
CKD	Chronic Kidney Disease
SGLT2	Sodium–Glucose Cotransporter-2
HF	Heart Failure
LVEF	Left Ventricular Ejection Fraction
ESRD	End-Stage Renal Disease
EMR	Electronic Medical Records
HCOs	Healthcare Organizations
ICD-10-CM	International Classification of Diseases, 10th Revision, Clinical Modification
RxNorm	Normalized Naming System for Generic and Branded Drugs
ATC	Anatomical Therapeutic Chemical Classification
PSM	Propensity Score Matching
HR	Hazard Ratio
CI	Confidence Interval
RR	Risk Ratio
OR	Odds Ratio
ER	Emergency Room
AKI	Acute Kidney Injury
BNP	B-type Natriuretic Peptide
NT-proBNP	N-terminal pro–B-type Natriuretic Peptide
HbA1c	Hemoglobin A1c
MR	Mineralocorticoid Receptor
RCTs	Randomized Controlled Trials
