# Supplementary material for: Comparative Effectiveness of Finerenone Versus SGLT2 Inhibitors in Patients with HFpEF and CKD: A Real-World Propensity-Matched TriNetX Analysis
Source: Biomedicines. 2026 May 14;14(5):1108. doi: 10.3390/biomedicines14051108 (PMC13204349; doi:10.3390/biomedicines14051108)

Supplemental Table S1. Codes and definitions used for cohort selection, exposures, and outcomes.

Variable	ICD 10 Code
**Base population**	
Chronic kidney disease, stage 1	N18.1
Chronic kidney disease, stage 2 (mild)	N18.2
Chronic kidney disease, stage 3 (moderate)	N18.3
Chronic kidney disease, stage 4 (severe)	N18.4
Chronic kidney disease, stage 5	N18.5
Chronic kidney disease, unspecified	N18.9
Chronic kidney disease (CKD)	ICD-9-CM-585
Diastolic (congestive) heart failure	I50.3
Other heart failure	I50.8
Heart failure, unspecified	I50.9
Systolic (congestive) heart failure	150.2
End stage renal disease	N18.6
Dependence on renal dialysis	Z99.2
**Medication**	
Finerenone	RxNorm 2562811
SGLT2 inhibitors	NLM ATC A10BK
**Laboratory**	
Left Ventricular Ejection Fraction (LVEF) (%) (at most 40.00 %)	TNX FINDING 2003
**Procedure**	
Hemodialysis - single evaluation	CPT:90935
Hemodialysis - repeated evaluation	CPT:90937
Unlisted dialysis procedure	CPT:90999
**Outcomes**	
All-Cause Mortality	Deceased
All-Cause Hospitalization/ER Visits	HL7V3.0:VisitType:EMER<br>HL7V3.0:VisitType:ACUTE<br>HL7V3.0:VisitType:IMP<br>HL7V3.0:VisitType:NONAC<br>HL7V3.0:VisitType:OBSENC
HF Hospitalization	ICD-10-CM-I50<br>HL7V3.0:VisitType:IMP
Hyperkalemia ≥5.5	TNX 9028

AKI	ICD-10-CM-N17
Creatinine ≥2.0	TNX 9024

Supplemental Table S2. Baseline characteristics of finerenone and SGLT2 inhibitor cohorts before and after propensity score matching.

Characteristic	Finerenone Group (N = 335)	SGLT2 Inhibitor Group (N = 48,296)	Std. Diff.	Finerenone Group (N = 333)	SGLT2 Inhibitor Group (N = 333)	Std. Diff.
	Before PSM			After PSM		
Demographics						
Age at Index (years), mean ± SD	72.0 ± 10.1	73.7 ± 10.7	0.163	72.0 ± 10.1	72.8 ± 11.1	0.069
Female	179 (53.4%)	24,165 (50.0%)	0.068	177 (53.2%)	172 (51.7%)	0.03
Male	156 (46.6%)	24,085 (49.9%)	0.066	156 (46.8%)	161 (48.3%)	0.03
Black or African American	39 (11.6%)	8,433 (17.5%)	0.166	39 (11.7%)	36 (10.8%)	0.029
White	193 (57.6%)	30,841 (63.9%)	0.128	193 (58.0%)	195 (58.6%)	0.012
American Indian or Alaska Native	10 (3.0%)	209 (0.4%)	0.198	10 (3.0%)	10 (3.0%)	<0.001
Native Hawaiian or Other Pacific Islander	10 (3.0%)	372 (0.8%)	0.164	10 (3.0%)	10 (3.0%)	<0.001
Asian	52 (15.5%)	2,543 (5.3%)	0.341	51 (15.3%)	47 (14.1%)	0.034
Other Race	10 (3.0%)	963 (2.0%)	0.064	10 (3.0%)	10 (3.0%)	<0.001
Unknown Race	35 (10.4%)	4,935 (10.2%)	0.008	35 (10.5%)	38 (11.4%)	0.029
Unknown Ethnicity	82 (24.5%)	12,312 (25.5%)	0.023	82 (24.6%)	79 (23.7%)	0.021
Not Hispanic or Latino	237 (70.7%)	33,792 (70.0%)	0.017	235 (70.6%)	241 (72.4%)	0.04
Hispanic or Latino	16 (4.8%)	2,192 (4.5%)	0.011	16 (4.8%)	13 (3.9%)	0.044
Comorbidities						
Essential (primary) hypertension	243 (72.5%)	35,081 (72.6%)	0.002	242 (72.7%)	236 (70.9%)	0.04
Type 2 diabetes mellitus	238 (71.0%)	30,420 (63.0%)	0.172	237 (71.2%)	238 (71.5%)	0.007
Overweight and obesity	101 (30.1%)	17,466 (36.2%)	0.128	100 (30.0%)	99 (29.7%)	0.007
Acute kidney failure	102 (30.4%)	14,269 (29.5%)	0.02	100 (30.0%)	104 (31.2%)	0.026
Chronic ischemic heart disease	143 (42.7%)	21,657 (44.8%)	0.043	141 (42.3%)	149 (44.7%)	0.048
Acute myocardial infarction	20 (6.0%)	4,780 (9.9%)	0.146	20 (6.0%)	21 (6.3%)	0.012
Cerebral infarction	23 (6.9%)	3,143 (6.5%)	0.014	23 (6.9%)	20 (6.0%)	0.037
Transient cerebral ischemic attacks and related syndromes	10 (3.0%)	1,201 (2.5%)	0.031	10 (3.0%)	11 (3.3%)	0.017
Atherosclerosis	47 (14.0%)	5,553 (11.5%)	0.076	47 (14.1%)	56 (16.8%)	0.075
Peripheral vascular disease, unspecified	37 (11.0%)	5,045 (10.4%)	0.019	37 (11.1%)	39 (11.7%)	0.019
Other chronic obstructive pulmonary disease	60 (17.9%)	11,061 (22.9%)	0.124	59 (17.7%)	77 (23.1%)	0.134
Obstructive sleep apnea	81 (24.2%)	12,208 (25.3%)	0.025	81 (24.3%)	86 (25.8%)	0.035

Iron deficiency anemia	40 (11.9%)	7,339 (15.2%)	0.095	39 (11.7%)	32 (9.6%)	0.068
Other anemias	92 (27.5%)	11,498 (23.8%)	0.084	91 (27.3%)	91 (27.3%)	<0.001
Fibrosis and cirrhosis of liver	19 (5.7%)	1,728 (3.6%)	0.1	19 (5.7%)	18 (5.4%)	0.013
Alcoholic liver disease	10 (3.0%)	379 (0.8%)	0.162	10 (3.0%)	10 (3.0%)	<0.001
Neoplasms	97 (29.0%)	11,884 (24.6%)	0.098	96 (28.8%)	89 (26.7%)	0.047
Tobacco use	10 (3.0%)	1,515 (3.1%)	0.009	10 (3.0%)	13 (3.9%)	0.049
Personal history of nicotine dependence	64 (19.1%)	11,673 (24.2%)	0.123	64 (19.2%)	54 (16.2%)	0.079
Alcohol related disorders	10 (3.0%)	1,243 (2.6%)	0.025	10 (3.0%)	10 (3.0%)	<0.001
Nicotine dependence	27 (8.1%)	4,559 (9.4%)	0.049	27 (8.1%)	30 (9.0%)	0.032
Other secondary pulmonary hypertension	24 (7.2%)	7,915 (16.4%)	0.289	24 (7.2%)	26 (7.8%)	0.023
Medications						
Beta blockers	174 (51.9%)	28,114 (58.2%)	0.126	174 (52.3%)	179 (53.8%)	0.03
Diuretics	166 (49.6%)	31,241 (64.7%)	0.309	166 (49.8%)	170 (51.1%)	0.024
Antiarrhythmics	126 (37.6%)	19,775 (40.9%)	0.068	125 (37.5%)	116 (34.8%)	0.056
ACE inhibitors	52 (15.5%)	8,991 (18.6%)	0.082	51 (15.3%)	53 (15.9%)	0.017
Warfarin	10 (3.0%)	2,369 (4.9%)	0.099	10 (3.0%)	10 (3.0%)	<0.001
Rivaroxaban	15 (4.5%)	2,461 (5.1%)	0.029	15 (4.5%)	15 (4.5%)	<0.001
Apixaban	58 (17.3%)	9,898 (20.5%)	0.081	58 (17.4%)	60 (18.0%)	0.016
Dabigatran etexilate	0 (0%)	283 (0.6%)	0.109	0 (0%)	0 (0%)	—
Aspirin	98 (29.3%)	16,237 (33.6%)	0.094	97 (29.1%)	106 (31.8%)	0.059
Clopidogrel	41 (12.2%)	6,122 (12.7%)	0.013	40 (12.0%)	39 (11.7%)	0.009
Angiotensin II inhibitors	148 (44.2%)	15,112 (31.3%)	0.268	146 (43.8%)	155 (46.5%)	0.054
Valsartan	42 (12.5%)	4,518 (9.4%)	0.102	40 (12.0%)	44 (13.2%)	0.036
Sacubitril	11 (3.3%)	1,828 (3.8%)	0.027	11 (3.3%)	10 (3.0%)	0.017
Antilipemic agents	195 (58.2%)	29,194 (60.4%)	0.046	193 (58.0%)	206 (61.9%)	0.08
Oral hypoglycemic agents	81 (24.2%)	12,683 (26.3%)	0.048	81 (24.3%)	71 (21.3%)	0.072
Laboratory Values						
Hemoglobin (g/dL)	12.1 ± 2.3	11.8 ± 2.3	0.107	12.1 ± 2.3	12.2 ± 2.3	0.025
Hemoglobin 0–0 g/dL	266 (79.4%)	38,473 (79.7%)	0.006	264 (79.3%)	261 (78.4%)	0.022
Natriuretic peptide B (pg/mL)	292.0 ± 396.7	880.0 ± 2580.4	0.319	292.0 ± 396.7	1580.5 ± 5205.7	0.349
Natriuretic peptide B 0–0 pg/mL	55 (16.4%)	12,676 (26.2%)	0.242	55 (16.5%)	58 (17.4%)	0.024
Natriuretic peptide B prohormone N-terminal (pg/mL)	2144.0 ± 2645.1	3642.3 ± 6140.2	0.317	2144.0 ± 2645.1	2131.4 ± 2834.4	0.005
Natriuretic peptide B prohormone N-terminal 0–0 pg/mL	55 (16.4%)	10,273 (21.3%)	0.124	55 (16.5%)	51 (15.3%)	0.033
Hemoglobin A1c (%)	6.9 ± 1.3	7.2 ± 1.7	0.22	6.9 ± 1.3	7.2 ± 1.5	0.239
Hemoglobin A1c 0–0 %	213 (63.6%)	28,400 (58.8%)	0.098	212 (63.7%)	223 (67.0%)	0.069

BMI (kg/m²)	32.7 ± 8.9	33.2 ± 8.5	0.055	32.7 ± 8.9	32.8 ± 8.7	0.002
BMI 0–0 kg/m²	246 (73.4%)	35,235 (73.0%)	0.011	244 (73.3%)	237 (71.2%)	0.047
Blood Pressure, Systolic (mmHg)	133.2 ± 22.1	131.3 ± 21.5	0.088	133.1 ± 22.1	132.1 ± 19.9	0.051
Blood Pressure, Systolic 0–0 mmHg	268 (80.0%)	39,420 (81.6%)	0.041	266 (79.9%)	266 (79.9%)	<0.001
Blood Pressure, Diastolic (mmHg)	70.2 ± 12.6	70.9 ± 13.4	0.054	70.2 ± 12.7	71.4 ± 13.4	0.088
Blood Pressure, Diastolic 0–0 mmHg	268 (80.0%)	39,414 (81.6%)	0.041	266 (79.9%)	266 (79.9%)	<0.001
Creatinine (mg/dL)	1.6 ± 0.6	2.7 ± 12.0	0.129	1.6 ± 0.6	2.3 ± 11.6	0.094
Creatinine 0–0 mg/dL	276 (82.4%)	40,117 (83.1%)	0.018	274 (82.3%)	274 (82.3%)	<0.001

Supplementary Figure S1: Propensity Score Matching

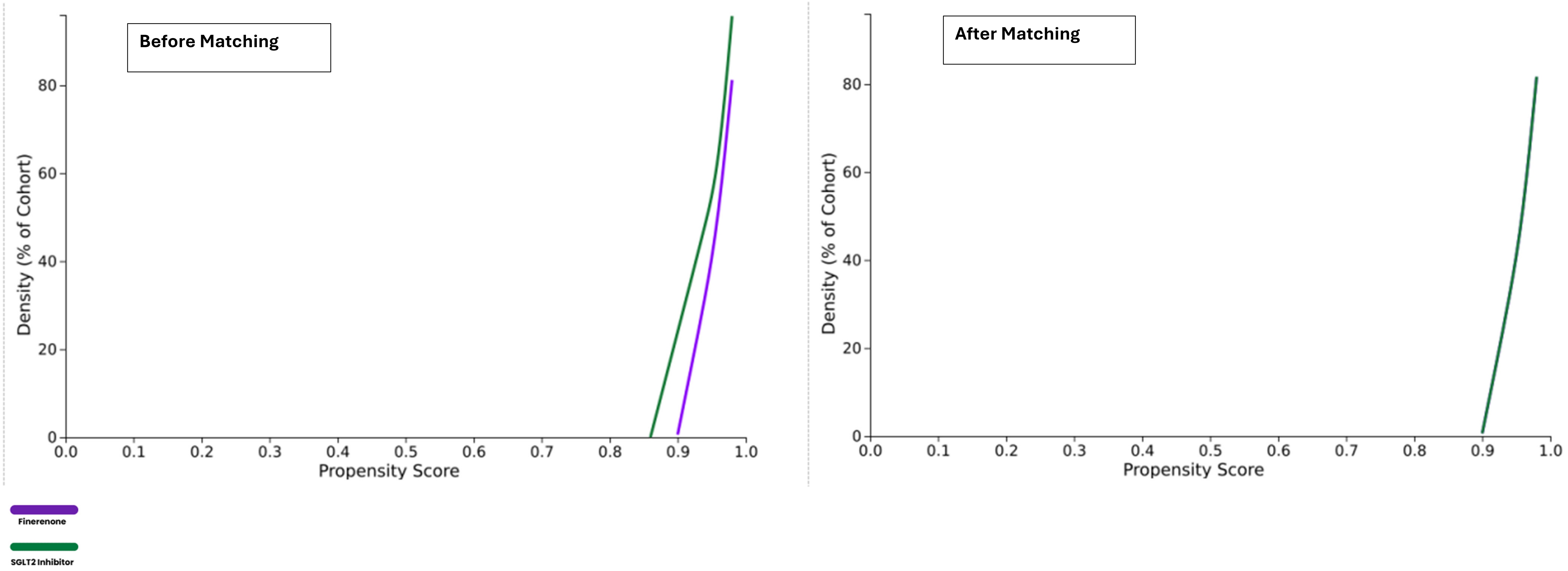

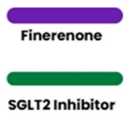

Supplementary Figure S2: Kaplan–Meier curves for All-Cause Hospitalization/ER Visits at 6-Months and 1-Year Follow-up.

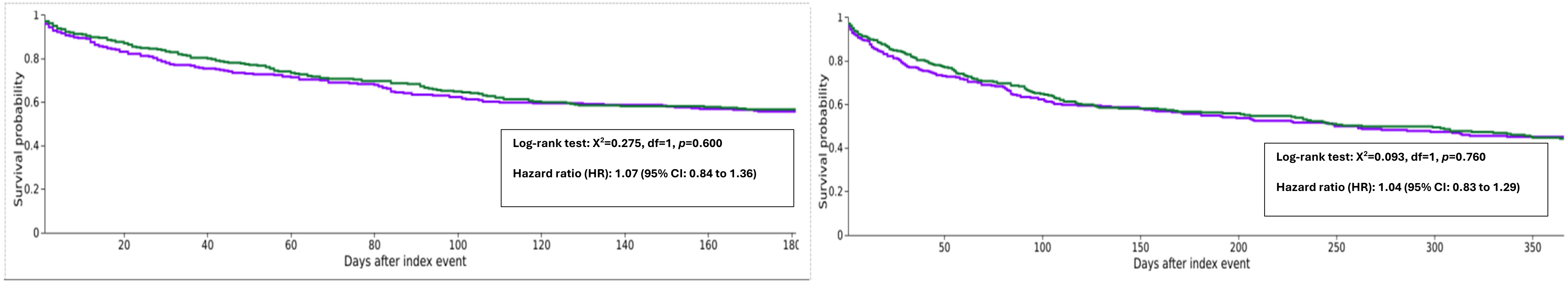

Supplementary Figure S3: Kaplan–Meier curves for Hyperkalemia ≥ 5.5 at 6-Months and 1-Year Follow-up.

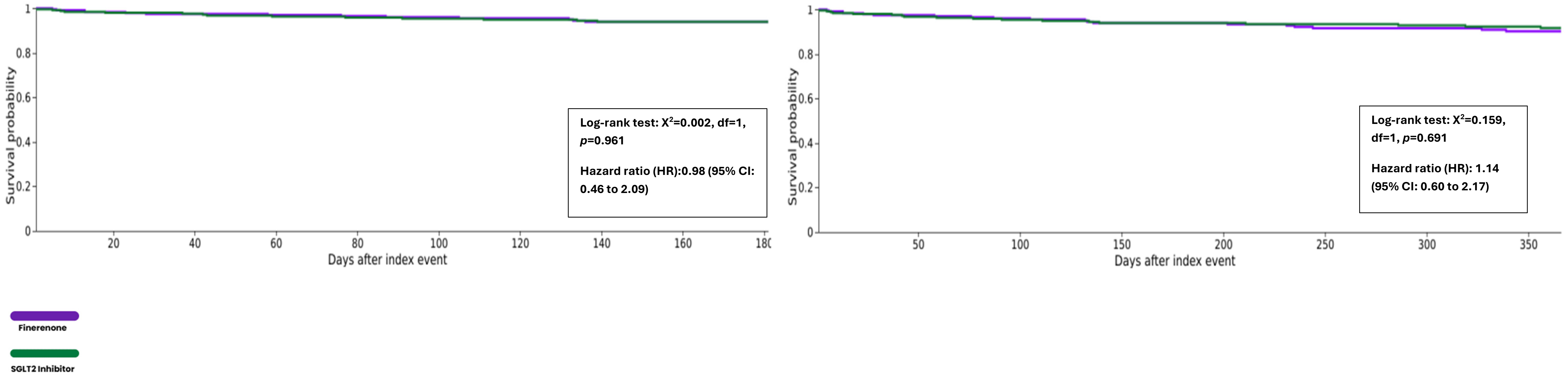

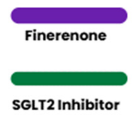

Supplementary Figure S4: Kaplan–Meier curves for Creatinine ≥ 2.0 at 6-Months and 1-Year Follow-up.

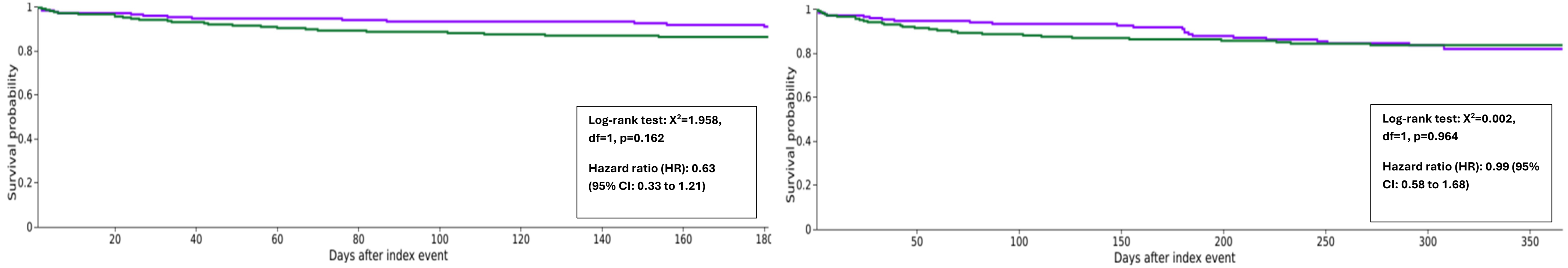

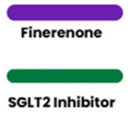

Supplementary Figure S5: Kaplan–Meier curves for Acute Kidney Injury (AKI) at 1-Year Follow-up.

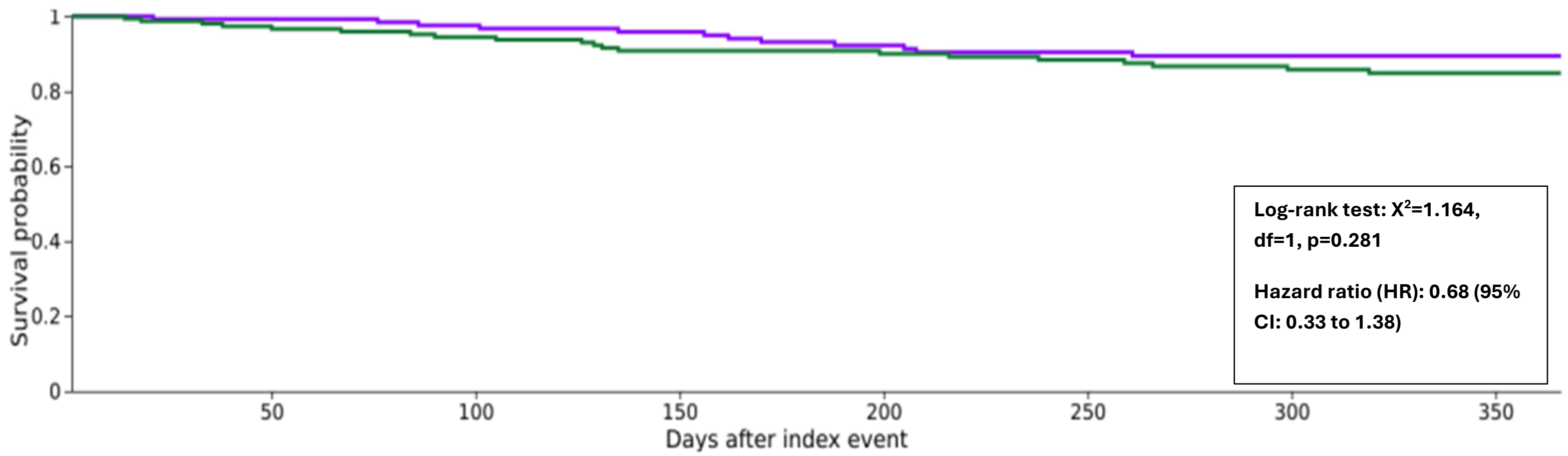

Supplement: Supplementary file 1 [file biomedicines-14-01108-s001.zip › biomedicines-4263336-supplementary.pdf]
